# Expression Profiling of mRNAs and Long Non-Coding RNAs in Aged Mouse Olfactory Bulb

**DOI:** 10.1038/s41598-017-02329-4

**Published:** 2017-05-18

**Authors:** Ming Wang, Wei Liu, Jian Jiao, Jingyun Li, Chengshuo Wang, Luo Zhang

**Affiliations:** 1 Department of Otolaryngology, Head and Neck Surgery, Beijing TongRen Hospital, Capital Medical University, Beijing, 100730 China; 2 Beijing Key Laboratory of Nasal Diseases, Beijing Institute of Otolaryngology, Beijing, 100005 China; 3 Department of Allergy, Beijing TongRen Hospital, Capital Medical University, Beijing, 100730 China; 4 The National Laboratory of Medical Molecular Biology, Institute of Basic Medical Sciences, Chinese Academy of Medical Sciences and Peking Union Medical College, Beijing, 100005 China

## Abstract

Age-related decline in olfactory function affects the quality of life in elderly people and also potentially represents an early clinical symptom of neurodegenerative disorder. Olfactory bulb (OB) plays a central role in olfactory information transmitting and signal processing. The mechanisms underlying this impairment remain unclear. In the current study, microarray was used to investigate differentially expressed protein coding genes (PCGs) and long non-coding RNAs (lncRNAs) in OBs from three groups of mice of different ages (2 months-old young adults, 6 months-old mature adults and 20 months-old aged adults), for their potential roles in olfactory impairment. Gene Ontology and pathway analysis results showed that the differentially expressed PCGs in the OBs from aged mice were mainly associated with signal transduction, regulation of gene expression and cellular microenvironment. Similarly, gene set enrichment analysis identified two differentially and inversely expressed lncRNAs (NONMMUT004524 and NONMMUT000384), both of which were significantly associated with neuroactive ligand-receptor interaction pathway in the OBs of aged mice. These findings suggest that a decline of olfactory function in aged mice may be linked to differential expression of specific lncRNAs and their potentially adverse effects on the neuroactive ligand-receptor interaction pathway in the OB.

## Introduction

Olfactory function decline is associated with normal aging, leading to a lower quality of life, impairment of food intake and increased risk for injury due to undetected gas leakage leading to explosion^1^. In addition, the reduction of olfactory function potentially represents an important early clinical symptom of neurodegenerative disorders such as Parkinson’s disease and Alzheimer’s disease^2^. Functional olfactory deficits during aging include increased threshold for odor detection, reduced ability in odor identification and discrimination, and impairment in olfactory associative learning and memory^3–5^. Although many studies have described age-related olfactory impairment, relatively little is known about the underlying molecular and cellular mechanisms.

The olfactory system is composed of well-defined peripheral and central structures. Olfactory sensory neurons (OSN) in the olfactory epithelium convert external signals to electrical activity and project to the olfactory bulb (OB), the first central structure of the olfactory pathway in the brain, which contain OB principal neurons that subsequently project to higher structures via the lateral olfactory tract^6^. The OB plays a central role in olfactory information transmission and signal processing including discrimination^7^, learning^3^ and memory^8^, and changes in the OB, especially during aging have been shown to contribute to impaired olfaction. An investigation of age-related changes in volume of the OB in 125 individuals aged 19 to 79 years, using magnetic resonance imaging (MRI), demonstrated that OB volumes were significantly correlated in relation to olfactory function and that OB volumes decreased with age^9^. Similarly, studies in animals have also reported an increased volume of the OB in adult rats and a proportional decreased volume of the OB laminae in aged rats^10^. Although cellular changes in the OB; including disrupted glomerular targeting of odorant receptors^11^, atrophy of mitral cell bodies^10^, reduced newborn interneurons^12^ and morphological change of astrocytes^13^; have been shown to be associated with declined olfactory function, the molecular mechanisms underlying these age-related changes of OB are still unknown.

Long noncoding RNAs (lncRNAs), which are transcripts longer than 200 nucleotides with no open reading frames, affect biological processes such as regulation of gene expression, genomic imprinting, cell survival, cell cycle and metabolism^14, 15^. Furthermore, recent studies have indicated that altered expression of lncRNAs is closely associated with aging and diseases such as cancer, neurodegenerative disorders and cardiovascular diseases^16, 17^. Moreover, lncRNAs have been implicated to play important roles in the development of sensory organs and related diseases^18, 19^, although any role of lncRNAs in olfactory function or related diseases remain unknown. Thus, in view of these findings, the aim of the present study was to explore the putative molecular mechanisms underlying age-related olfactory impairment, by investigating the differentially expressed (DE-) protein coding genes (PCGs) and lncRNAs in the OB of mice of different ages. Olfactory bulb of young adult (2 month old), mature (6 month old) and aged (20 month old) males were assessed by microarray to obtain expression profiles of both mRNAs and lncRNAs. Part of the DE-PCGs and DE-lncRNAs were validated by real-time quantitative PCR in external tissue. The functional annotation of DE-PCGs and possible functions of DE-lncRNAs were further inferred.

## Methods

### Experimental animals

Three groups of male C57BL/6 mice; consisting of nine 2-month-old young animals (weight 22 g to 24 g), 6-month-old mature animals (weight 30 g to 32 g), or 20-month-old aged animals (weight 42 g to 45 g); were obtained from Vital River Laboratory Animal Technology Co., Ltd (Beijing, China). The mice were housed at 22 ± 2 °C under a 12 h light–dark cycle in bioclean units with free access to water and standard nutrition. Each animal was euthanized with CO_2_ and microdissection was performed to isolate the OBs. Immediately following tissue harvesting, the tissues were snap-frozen in liquid nitrogen. OBs from three animals in each group were used for microarray analysis and from the remaining six animals were used for validation by real-time quantitative reverse transcription- polymerase chain reaction (qRT-PCR).

All study protocols were implemented in accordance with NIH guidelines and were approved by the Animal Care and Use Committee of Capital Medical University.

### RNA extraction

Total RNA was extracted from mouse OB using Trizol reagent (Invitrogen, Carlsbad, CA) according to manufacturer’s instructions, and digested with DNase I at 37 °C for 15 min to remove any contaminating DNA. The RNA was cleaned up with RNeasy Kit (Qiagen, Hilden, Germany) and RNA concentration was measured with ND-1000 spectrophotometer (NanoDrop Technologies, Wilmington, DE). RNA quality was evaluated using 1% formaldehyde denaturing gel electrophoresis. The samples with bright bands of ribosomal 28S and 18S RNA in a ratio >1.5: 1 were used for microarray analysis.

### Microarray Analysis

The GeneChip Mouse Transcriptome Array 1.0 (also known as Clariom D assays; Affymetrix, Thermo Fisher Scientific Inc.) was used to provide a detailed analysis of the transcriptome, covering transcripts of 26, 336 mRNAs and 39, 595 lncRNAs. Briefly, 100 ng of total RNA from each of three samples originally assigned for microarray analysis were used to generate amplified and biotinylated sense-strand cDNA from the entire expressed genome according to the GeneChip WT PLUS Reagent Kit User Manual (P/N 703174, Affymetrix Inc., Santa Clara, CA). cDNA was hybridized to GeneChip Mouse Transcriptome Array 1.0 for 16 hours in a 45 °C incubator, rotated at 60 rpm. After hybridization, the microarrays were washed, and then stained using the Fluidics Station 450 followed by scanning with the Affymetrix GeneChip Scanner 3000 7G, according to manufacturer’s instructions.

Raw data was normalized using the quantile normalization of robust multiarray average (RMA) method (performed at the individual probe level) in the Expression Console 1.4 (software provided by Affymetrix). Significance Analysis of Microarrays (SAM) was used to identify transcripts that were differentially expressed. PCGs and lncRNAs were determined to be differentially expressed when the ANOVA *p*-value was <0.05 and fold change was greater than 1.5. Hierarchical cluster analysis of differentially expressed transcripts was performed using cluster 3.0^20^ with average linkage and uncentred Pearson correlation. The log2-scaled expression values were centred on the median before performing hierarchical clustering. The chromosomal distribution of DE-PCGs and DE-lncRNAs was identified and showed as circular maps.

### Quantitative Real-time PCR

Microarray data were validated by qRT-PCR. Four lncRNAs and two mRNAs were randomly selected for validation in the six samples of each group, assigned to for qRT-PCR analysis. The primers were designed online using Primer3 and evaluated by NCBI BLAST (https://www.ncbi.nlm.nih.gov/tools/primer-blast) to ensure specificity. All the primer sequences are shown in Supplementary File 1. PCR amplifications were performed using SYBR Green Premix (Takara Bio Inc.) according to manufacturer’s instructions and qRT-PCR analysis and data collection were carried out on the ABI 7500 qPCR system. For each lncRNA or mRNA, qRT-PCR reactions were performed in triplicate, with glyceraldehyde-3-phosphate dehydrogenase (GAPDH) as internal control gene for normalization. The relative expression was calculated using the 2−ΔΔCT method.

### Gene ontology (GO) and Pathway analysis

GO terms were used to describe gene and gene product attributes. DE-PCGs were loaded into DAVID Bioinformatics Resources^21^ (https://david.ncifcrf.gov) for GO enrichment analysis. Values of *p* < 0.05 were considered to be statistically significant; with lower *p*-values indicating greater significance of the GO terms enrichment in DE-PCGs.

Pathway mapping was used to predict the main pathways of the altered PCGs. All altered PCGs with *p*-value < 0.05 were loaded into KOBAS 2.0 (KEGG Orthology Based Annotation System2.0)^22^; which incorporates pathway information from 5 pathway databases (KEGG PATHWAY, PID, BioCyc, Reactome and Panther); to identify significantly enriched pathways. Values of *p* < 0.05 were again considered to be statistically significant; with the lower *p*-values indicating greater significance of the pathway.

### Prediction of lncRNA function

Most of the lncRNAs have not yet been functionally annotated in current databases. One way to predict lncRNA function is based on the functional annotations of their related PCGs. Pearson correlation coefficient was calculated between the expression levels of DE-lncRNAs and all the PCGs. We defined PCGs potentially co-expressed with the lncRNA with Pearson correlation coefficient >0.8 or <−0.8, and *p*-value < 0.05. The gene set enrichment analysis (GSEA)^23^ was subsequently used to determine if the co-expressed PCGs of a given gene set were generally associated with a certain lncRNA. The significance threshold was set at *p*-value < 0.05 and a false discovery rate (FDR) cutoff of 25%. A positive/negative enrichment score indicated a positive/ negative correlation between the lncRNA and the pathway.

### Statistical analysis

The SPSS 17.0 software (SPSS Inc., Chicago, IL, USA) was used to analyze all data. Differences in lncRNA or PCG expression between two groups were compared by independent samples t-test and differences were considered significant at *p*-value < 0.05.

## Results

### Identification of DE-PCGs and DE-lncRNAs in Aged Mouse OB

**Figure 1:**
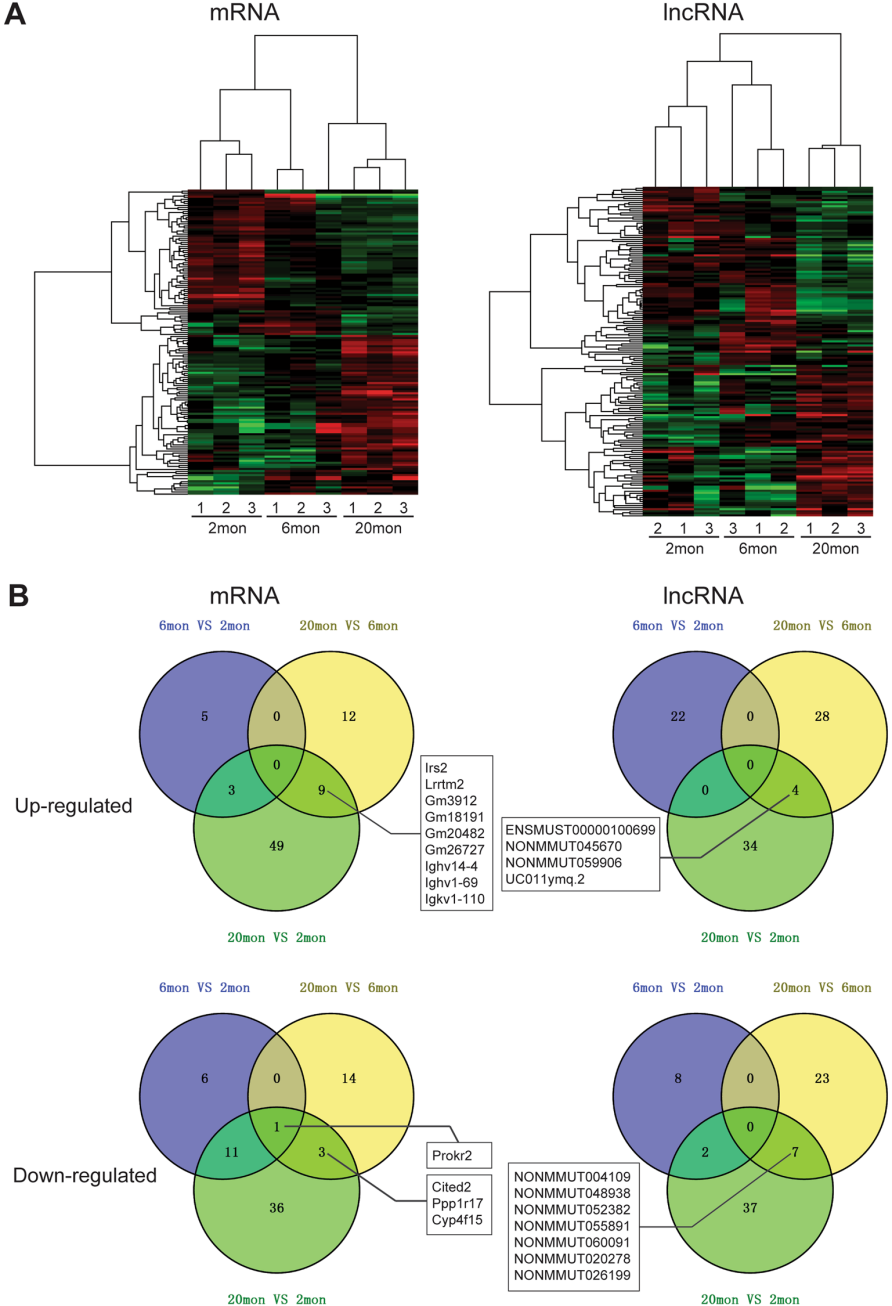
DE-PCGs and DE-lncRNAs in aged mouse OB. (**A**) Hierarchical clustering of DE-lncRNAs and DE-mRNAs in aged (20-month-old) versus young adult (2-month-old), aged versus mature (6-month-old), and mature versus young adult mouse OB (ANOVA *p*-value < 0.05 and fold change >1.5). (**B**) Venn diagrams depicting up-regulated and down-regulated mRNAs and lncRNAs in mouse OB of different ages. The number of DE-PCGs or DE-lncRNAs is marked in the corresponding areas, and common DE-PCGs and DE-lncRNAs shared by aged versus young adult and aged versus mature mice are listed.

The microarray data for OBs from three animals in each age group detected a total of 26, 336 mRNAs and 39, 595 lncRNAs transcripts. DE-PCGs and DE-lncRNAs, identified based on fold change (>1.5 or <−1.5) plus a significant *p*-value (<0.05). Following SAM analysis, the DE-mRNAs and DE-lncRNAs were subjected to hierarchical clustered as shown in Fig. 1A; with the red and green color coding representing the level of expression above and below the relative expression, respectively. Figure 1B shows the up-regulated and down-regulated mRNAs and lncRNAs in mouse OB of different ages. Overall, there were 112 DE-PCGs (61 up- and 51 down-regulated) and 84 DE-lncRNAs (38 up- and 46 down-regulated) in aged mice versus young adult mice. Similarly, 39 DE-PCGs (21 up- and 18 down-regulated) and 62 DE-lncRNAs (32 up- and 30 down-regulated) were found in aged mice versus mature mice. Furthermore, the two sets of DE-PCGs and DE-lncRNAs shared 13 up-regulated transcripts (9 PCGs and 4 lncRNAs) and 11 down-regulated common transcripts (4 PCGs and 7 lncRNAs) (Fig. 1B). Interestingly, only 26 PCGs (8 up- and 18 down-regulated) and 32 lncRNAs (22 up- and 10 down-regulated) were differentially expressed in mature mice versus young adult mice. More detailed information of DE-PCGs and DE-lncRNAs in mouse OB of different ages is provided in Supplementary File 2.

We further analyzed the chromosomal distribution of DE-PCGs and DE-lncRNAs. The DE-PCGs and DE-lncRNAs of mature versus young adult mice revealed random distribution across distinct chromosomes (Supplementary Figure 1). In contrast, the DE-PCGs and DE-lncRNAs of both aged versus young adult group and aged versus mature group showed maximum distribution on chromosome 7, accounting for 13.3% and 17.8% of total differentially expressed transcripts, respectively (Supplementary Figures 2 and 3).

### Validation of Microarray Data by qRT-PCR

**Figure 2:**
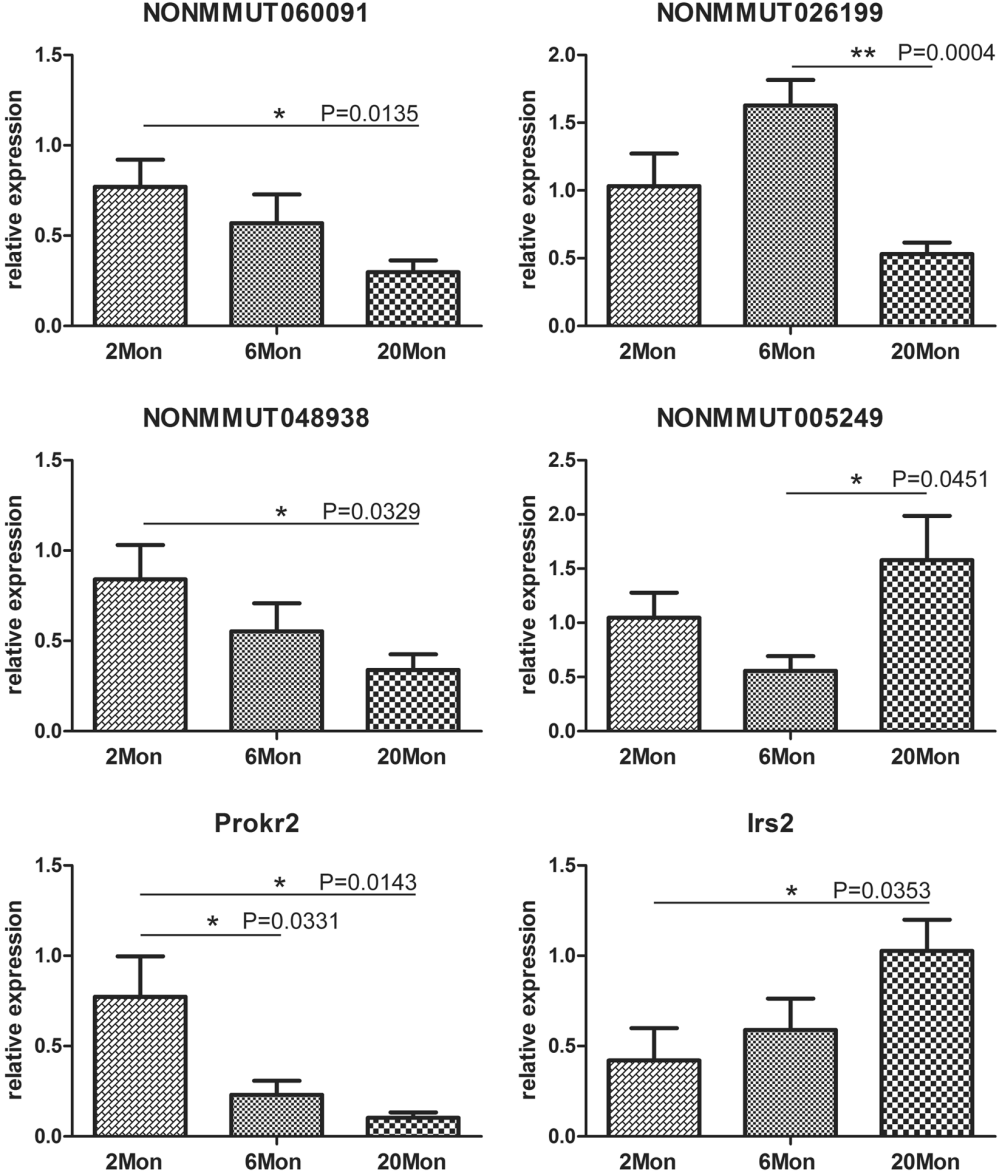
qRT-PCR validation of lncRNAs and mRNAs expression. Each sample was analysed in triplicate and GAPDH was used as the reference gene. Bars represent S.E.M. *p-value < 0.05, **p-value < 0.01.

Assessment by qRT-PCR for the expression of six randomly selected transcripts (4 lncRNAs and 2 mRNAs) in OB samples from 6 mice in each age group demonstrated similar trends and highly consistent data with the microarray data for each transcript (Fig. 2 and Supplementary Figure 4). LncRNA- NONMMUT060091, lncRNA- NONMMUT048938 and PCG- Prokr2 trended towards down-regulation with age and were significantly down-regulated in the aged group, compared to the young adult group (*p*-value < 0.05). In contrast, PCG- Irs2 showed significantly greater up-regulation in aged mice compared to the young adult mice (*p*-value < 0.05). LncRNA- NONMMUT026199 and lncRNA- NONMMUT005249 were significantly up-regulated and down-regulated, respectively, in aged mice compared with mature mice (*p*-value < 0.05).

### Signatures of DE-PCGs and DE-lncRNAs

**Figure 3:**
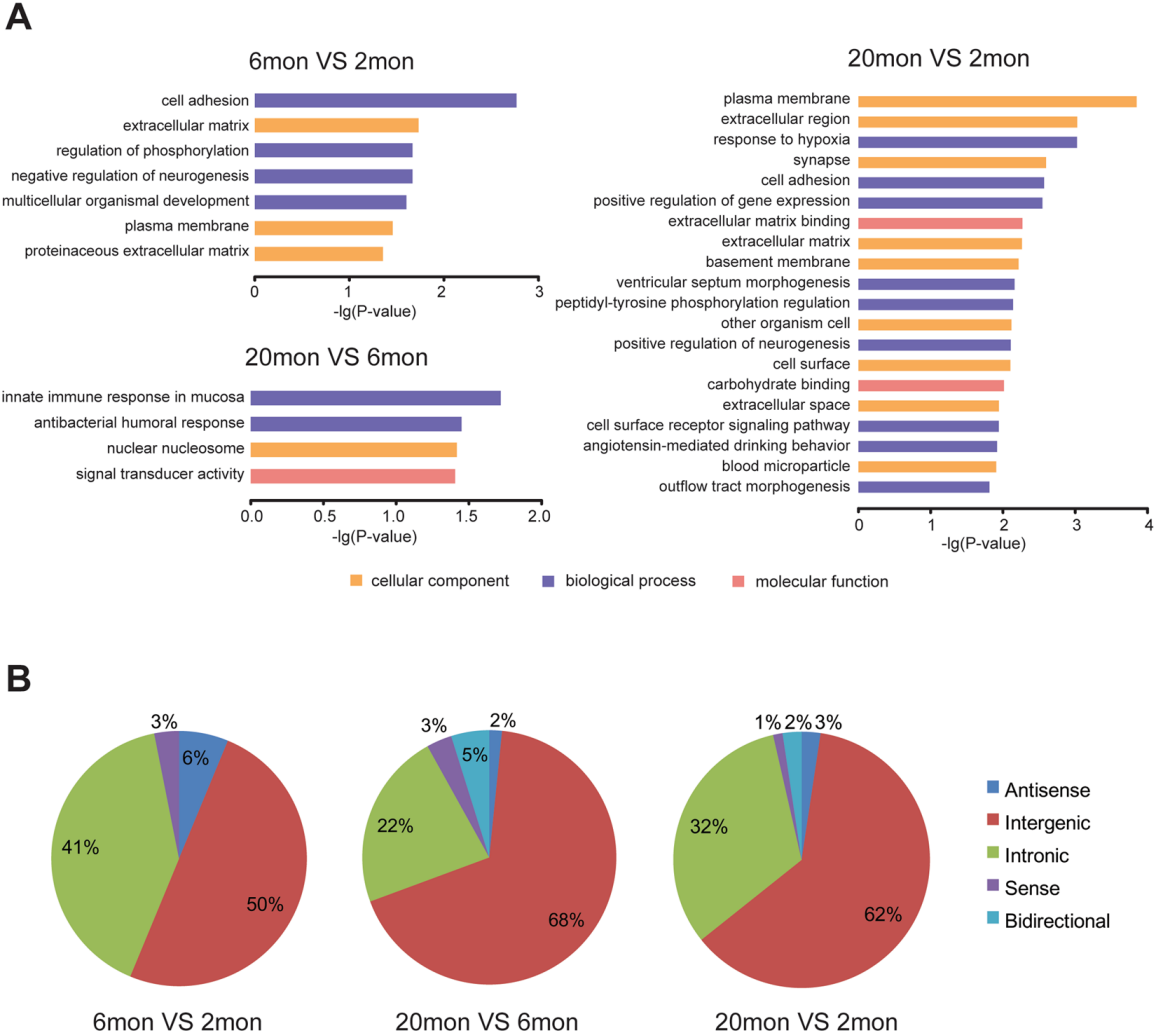
Signatures of DE-PCGs and DE-lncRNAs. (**A**) Gene ontology enrichment analysis was performed by DAVID for DE-PCGs in aged versus young adult, aged versus mature and mature versus young adult mouse OB. The resulting GO terms of cellular component, biological processes and molecular function with *p*-value < 0.05 were considered statistically significant and depicted. (**B**) Distribution of DE-lncRNAs according to their relationships with PCGs in genome (sense, antisense, intronic, bidirectional or intergenic). The percentage of each class are showed.

All the DE-PCGs were loaded into DAVID for GO enrichment analysis; with all or the top 20 significantly enriched GO terms including categories of biological process, molecular function, and cellular component (Fig. 3A). DE-PCGs of aged mice versus young adult mice were enriched mainly in plasma membrane, extracellular region and synapse of ontology ‘cellular component’; cell adhesion, regulation of gene expression and cell surface receptor signaling pathway of ontology ‘biological process’; extracellular matrix binding of ontology ‘molecular function’. Similarly, signal transducer activity of ontology ‘molecular function’ was enriched by DE-PCGs of aged mice versus mature mice. These results for GO terms infer that DE-PCGs in aged mouse OB might play important roles in regulating intercellular communication exerted by synapse, cell surface and extracellular matrix, as well as regulating signal transducer activity and gene expression.

One way to describe lncRNAs is to classify them according to genomic location. As indicated in Fig. 3B, we identified five categories of lncRNAs according to their relationships with PCGs in the genome; including sense, antisense, intronic, bidirectional and intergenic. In this regard, the intergenic lncRNAs comprised the largest subgroup of DE-lncRNAs, which was 62% of DE-lncRNAs of aged mice versus young adult mice and 68% of DE-lncRNAs of aged mice versus mature mice.

### Pathway analysis of altered PCGs

**Figure 4:**
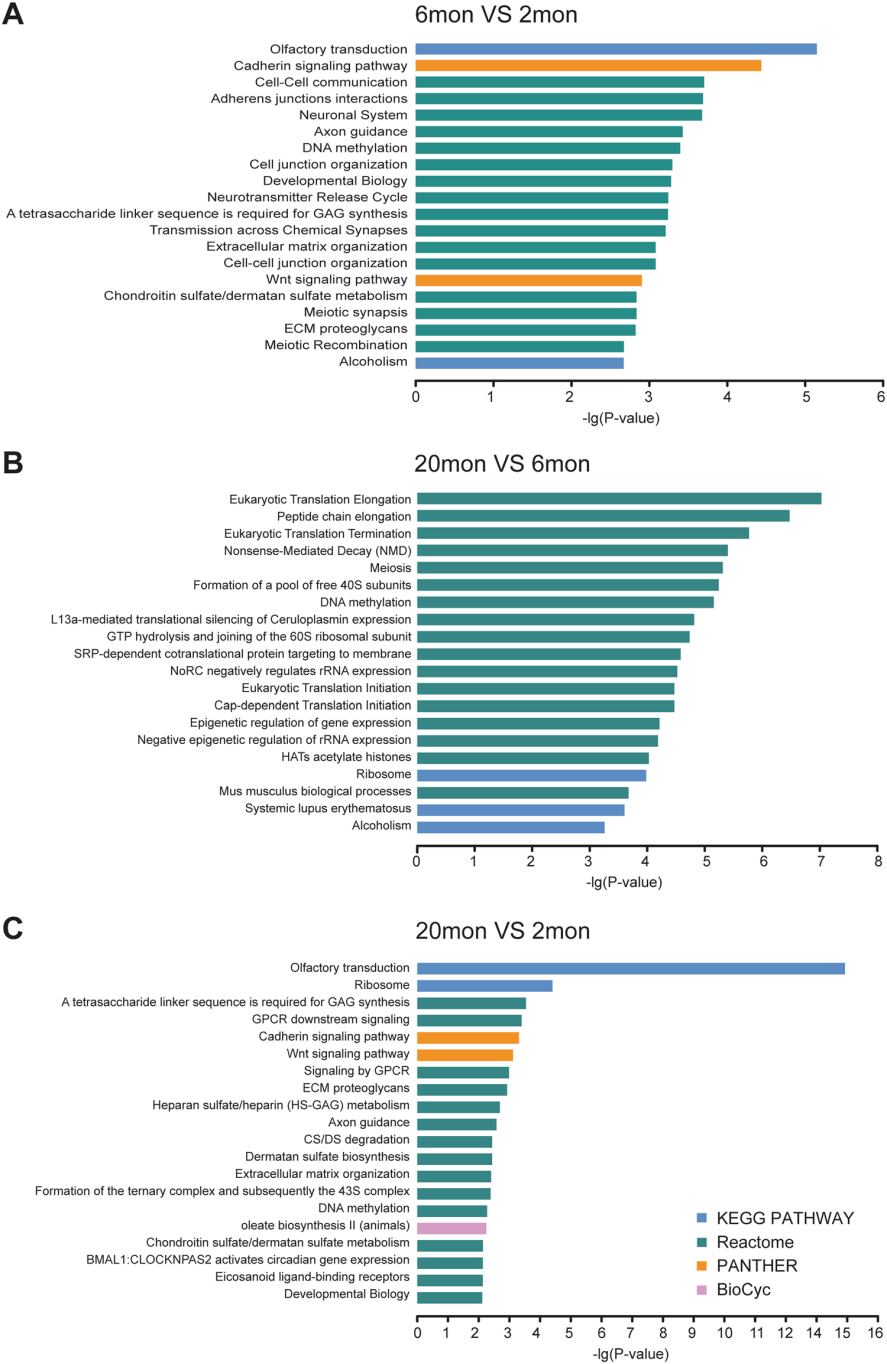
Pathway analysis of altered PCGs. Altered PCGs of mature versus young adult mice (**A**), aged versus mature mice (**B**), and aged versus young adult mice (**C**) were loaded into KOBAS 2.0 for assessment of enriched pathways. The top 20 significantly enriched pathways from pathway databases KEGG PATHWAY, BioCyc, Reactome and Panther were calculated when plotted as the −lg(*p*-values). Total significantly enriched pathways are shown in detail in Supplementary File 3.

KOBAS analysis showed that olfactory transduction, ribosome, axon guidance and signaling pathways like GPCR signaling were the most significantly enriched pathways for altered PCGs of aged group versus young adult group (Fig. 4C). In addition, the altered PCGs of aged group versus mature group were found to be mainly associated with eukaryotic translation progress (initiation, elongation and termination), meiosis, DNA methylation and ribosome (Fig. 4B). Overall pathway analysis indicated that aging affected a tissue specific pathway (olfactory transduction) the most in mice. Furthermore, some pathways such as GPCR signaling, ribosome and DNA methylation, which have been widely reported to be associated with the aging process, were also significantly enriched. Total significantly enriched pathways are shown in detail in Supplementary File 3.

### Prediction of lncRNA function

**Figure 5:**
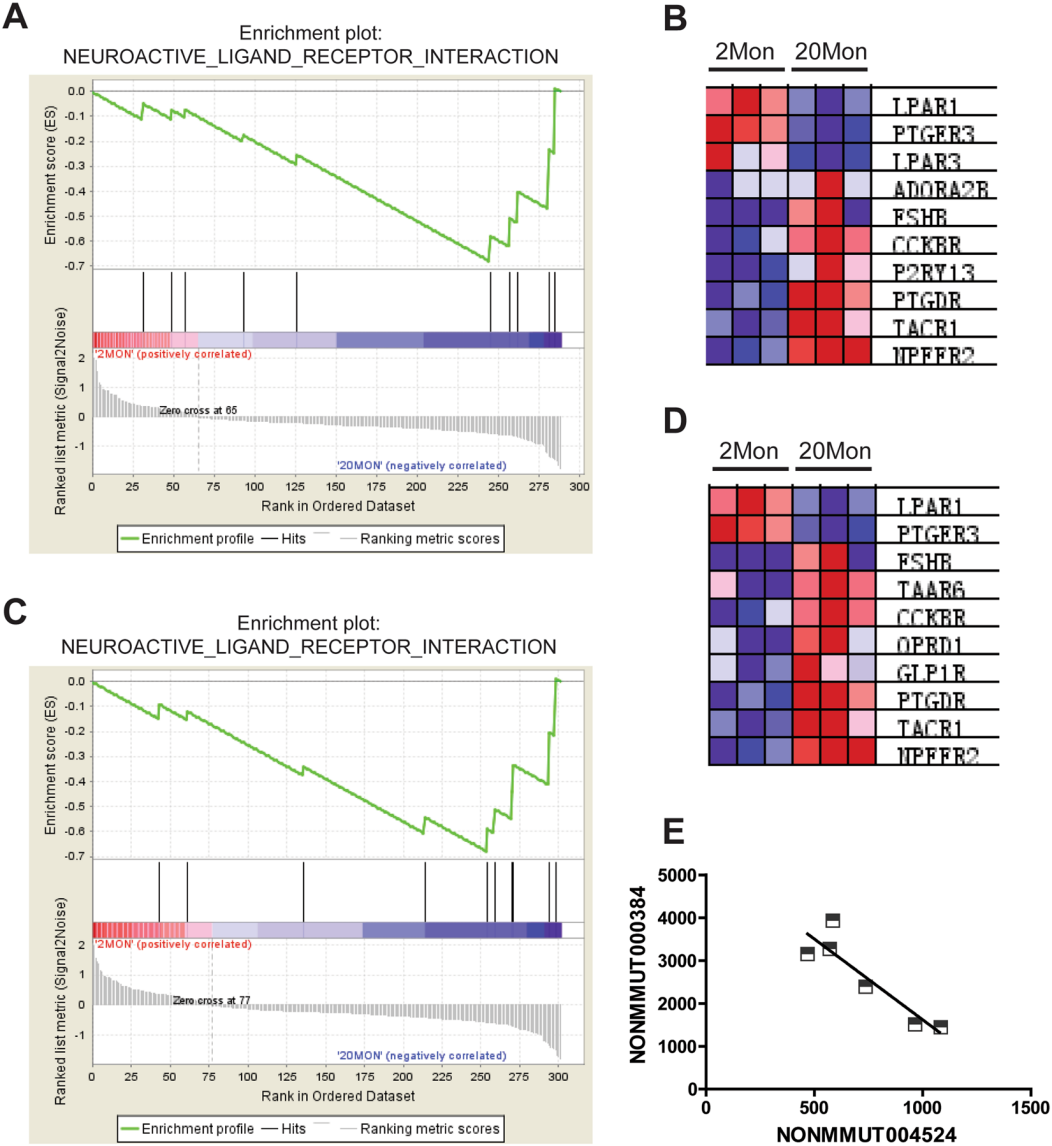
The most significant pathway associated with DE-lncRNAs in aged mouse OB. Pearson correlation coefficient was performed to determine correlations between DE-lncRNAs in aged mouse OB and all PCGs and then specific sets of genes with high correlation scores were used to annotate the function of the lncRNA by gene set enrichment analysis (GSEA) for KEGG pathways. (**A**,**B**) LncRNA-NONMMUT004524 (up-regulated in aged group) and (**C**,**D**) lncRNA-NONMMUT000384 (down-regulated in aged group) were found to be significantly associated with neuroactive ligand-receptor interaction pathway (*p*-value < 0.05, FDR < 25%). (**E**) Expression of NONMMUT004524 and NONMMUT000384 was significantly correlated when expressed conversely (*p*-value < 0.05, Pearson r = −0.906).

Specific sets of genes demonstrating statistically significant correlations between the expression of DE-lncRNAs and PCGs were further assessed by gene set enrichment analysis (GSEA) to annotate the function of DE-lncRNAs in aged mice. GSEA analysis showed that 17 lncRNAs were associated with 25 KEGG pathways in DE-lncRNAs of aged mice versus young adult mice (Supplementary File 4). Similarly, 17 lncRNAs were associated with 62 KEGG pathways in DE-lncRNAs of aged mice versus mature mice (Supplementary File 5). Among all the DE-lncRNAs in aged mice, DE-lncRNAs NONMMUT004524 and NONMMUT000384 were significantly associated with genes involved in the neuroactive ligand-receptor interaction pathway (Fig. 5A–D). Interestingly, the up-regulated expression of NONMMUT004524 was significantly correlated with down-regulated expression of NONMMUT000384 in aged versus young adult mice (Fig. 5E).

Furthermore, several other DE-lncRNAs of aged versus young adult mice were found to be generally associated with pathways involving focal adhesion, JAK-STAT signaling, Wnt signaling and neurotrophin pathways (Supplementary File 4). Similarly, several DE-lncRNAs of aged group versus mature group were generally associated with taste transduction pathway, ribosome, focal adhesion pathway and important metabolic pathways including fatty acid, amino acid, pyrimidine and propanoate metabolism (Supplementary File 5).

## Discussion

Age-related olfactory impairments affect the quality of life of elderly people and potentially represent an important early clinical symptom of neurodegenerative disorders. In this study, we investigated the DE-PCGs and DE-lncRNAs in aged mouse OB to explore the molecular mechanisms underlying age-related olfactory impairment. Our study demonstrated that differential expression of DE-PCGs were mainly associated with the olfactory transduction pathway and several signaling pathways, whereas differential expression of DE-lncRNAs; particularly NONMMUT004524 and NONMMUT000384; was associated with the neuroactive ligand-receptor interaction pathway. Furthermore, DE-PCGs and DE-lncRNAs of both aged versus young adult group and aged versus mature group showed maximum distribution on chromosome 7, which is of particular interest as associations between chromosome 7 and aging have been reported previously^24, 25^. To our knowledge, this is the first study to investigate the potential role of differential expression of lncRNAs and PCGs in the OBs of aged mice, and provide important pointers to the putative mechanisms underlying age-related olfactory impairment in humans.

Detailed analysis of the DE-PCGs in OB of aged mice indicated that almost all significantly enriched GO terms and pathways were related with the three categories of (1) signal transduction (such as olfactory transduction, synapse and cell surface receptor signaling pathway), (2) regulation of gene expression (including DNA methylation, transcription factors and ribosome proteins) and (3) cellular microenvironment (such as extracellular matrix, cell adhesion and ECM proteoglycans). These enriched GO terms and pathways may explain olfactory impairment, particularly as OB consist of multiple cell types including mitral cell, tufted cells, a diverse set of interneurons, OSN axons and glial cells^26^. Furthermore, regular intercellular communication and cellular microenvironment are crucial for precisely organized axonal connections and local neuronal circuits to deliver and process information^27^. As DNA methylation undergoes remodeling as aging progresses in various tissues of mice and humans^28^, our finding raises the possibility that the DNA methylation pathway may contribute to the dysregulation of gene expression during aging. Furthermore, in accordance with the findings of Zahn and colleagues^29^, our study also demonstrated a trend of increased expression of ribosome genes in the aged mice, which may related to the regulation of protein synthesis rates.

Our findings for the DE-PCGs in aged mouse OB are in accordance with those of others for various genes associated with aging. Taguchi and colleagues^30^ showed that less Irs2 signaling in aging brains could promote healthy metabolism and extend the life span of mice. Contrary to this finding, an increased expression level of Irs2 was found in our study of aged mouse OB, which might possibly lead to an increase in neuronal oxidative stress and mitochondrial dysfunction^31^. Cited2, a multi-stimuli responsive transactivator involved in cell senescence^32^, is down-regulated in aged mouse OB and may be related to neuron generation and connectivity^33^. Similarly, Lcn2 has been shown to be associated with aging, obesity^34^ and regulation of neurodegeneration^35^. Our finding of a decreased expression of Lcn2 in aged mouse OB, suggests that Lcn2 may play a role in controlling the aging of OB. Mitochondrial dysfunction is an important feature of normal aging and neurodegeneration, and mammalian cells have been shown to require Atf5 for maintaining mitochondrial activity during mitochondrial stress and to promote organelle recovery^36^. Similarly, a study of mitochondrial transporter protein Ucp2, which is associated with longevity in humans and mice, demonstrated that there was an accelerated aging process in Ucp2-deficient mice^37^. Consistent with these findings, microarray data from the present study also showed a decreased expression of Atf5 and Ucp2 in aged mice (Supplementary File 2).

Our study has demonstrated that the expression level of Prokr2 significantly decreased progressively from age 2 months, to 6 months to 20 months. Prokr2 is vital for olfactory bulb morphogenesis in mice and humans, and deficiency of prokr2 gene has been shown to lead to a loss of normal OB architecture or agenesis^38^. Evidence indicates that Prokr2 is necessary for the migration of new born neurons via the rostral migratory stream (RMS) and locate to the OB^39^. Our findings for Prokr2 suggest that progressively decreasing levels of Prokr2 with age may possibly contribute to decline in olfactory function through impairment of neurogenesis and location to OB.

Mechanistic studies of the aging process have provided increasing evidence that non-coding RNAs are important players in aging^16, 40^. We have previously shown that an abnormally low expression of microRNA-16 could potentially lead to APP protein accumulation in senescence-accelerated mouse prone 8 (SAMP8) mice^41^. Evidence from other studies shows that lncRNAs influence the molecular processes which underlie age associated phenotypes, such as telomere attrition, epigenetic alterations, cellular senescence, stem cell exhaustion, and altered intercellular communication^14^. For instance, lncRNA TERC, the essential RNA component of the telomerase enzyme complex, plays a role in the maintenance of telomere length and prevention of premature senescence and aging^42^. Similarly, lncRNA antisense-Uchl1 is involved in brain function and neurodegenerative diseases by increasing UCHL1 protein synthesis^43^.

In the present study, a total of 84 lncRNAs were found to be differentially expressed in aged mice compared to young adult mice, of which DE-lncRNAs NONMMUT004524 and NONMMUT000384 were most significantly associated with the neuroactive ligand-receptor interaction pathway. The neuroactive ligand-receptor interaction pathway consists of a variety of signaling molecules including many types of neuroreceptors, which are located on plasma membranes and involved in the transduction of signals from the extracellular environment into cells^44^. In the context of the present study, almost all the PCGs used to annotate the function of lncRNA NONMMUT004524 (P2ry13, Ptger3, Cckbr, Adora2b, Tacr1, Taar8b, Ptgdr, Npffr2, Lpar3, Lpar1, Taar4, Fshb) and NONMMUT000384 (Taar6, Ptger3, Cckbr, Tacr1, Ptgdr, Npffr2, Lpar1, Taar4, Fshb, Glp1r, Oprd1) are class A rhodopsin-like G protein-coupled receptors (GPCRs) of the neuroactive ligand-receptor interaction pathway. Given the changes observed in the expression and activity of GPCRs during aging, these receptors appear to be directly involved in aging and certain age-related pathologies^45^. Indeed, one study has demonstrated that lncRNA 17A was up-regulated in cerebral tissues of patients with Alzheimer disease and controlled the alternate splicing of G-protein coupled receptor 51^46^. Collectively, these findings suggest that lncRNA and GPCRs may be linked with age-associated decline in OB function.

In conclusion, our study has indicated that several PCGs and lncRNAs are expressed differentially in the OB of aged mice, compared to younger mice. Furthermore, a decline in olfactory function in aged mice may be linked to a differential and inverse expression of lncRNAs NONMMUT004524 and NONMMUT000384 and a potentially adverse effect of the lncRNAs on the neuroactive ligand-receptor interaction pathway in the OB of aged mice. However, further studies exploring the direct effects of these DE-lncRNAs in DE-lncRNA-deficient mice are required to substantiate these findings, and lead to a better understanding of the mechanisms underlying age-related olfactory impairment.

## Electronic supplementary material

Supplementary figures

Supplementary File 1

Supplementary File 2

Supplementary File 3

Supplementary File 4

Supplementary File 5
