# Supplementary material for: Expression Profiling of mRNAs and Long Non-Coding RNAs in Aged Mouse Olfactory Bulb
Source: Sci Rep. 2017 May 18;7:2079. doi: 10.1038/s41598-017-02329-4 (PMC5437011; doi:10.1038/s41598-017-02329-4)
Supplement: Supplementary file 1 — Supplementary figures [file 41598_2017_2329_MOESM1_ESM.pdf]

**Expression Profiling of mRNAs and Long Non-Coding RNAs in Aged Mouse Olfactory Bulb**

Ming Wang, Wei Liu, Jian Jiao, Jingyun Li, Chengshuo Wang, Luo Zhang

**Supplementary Materials**

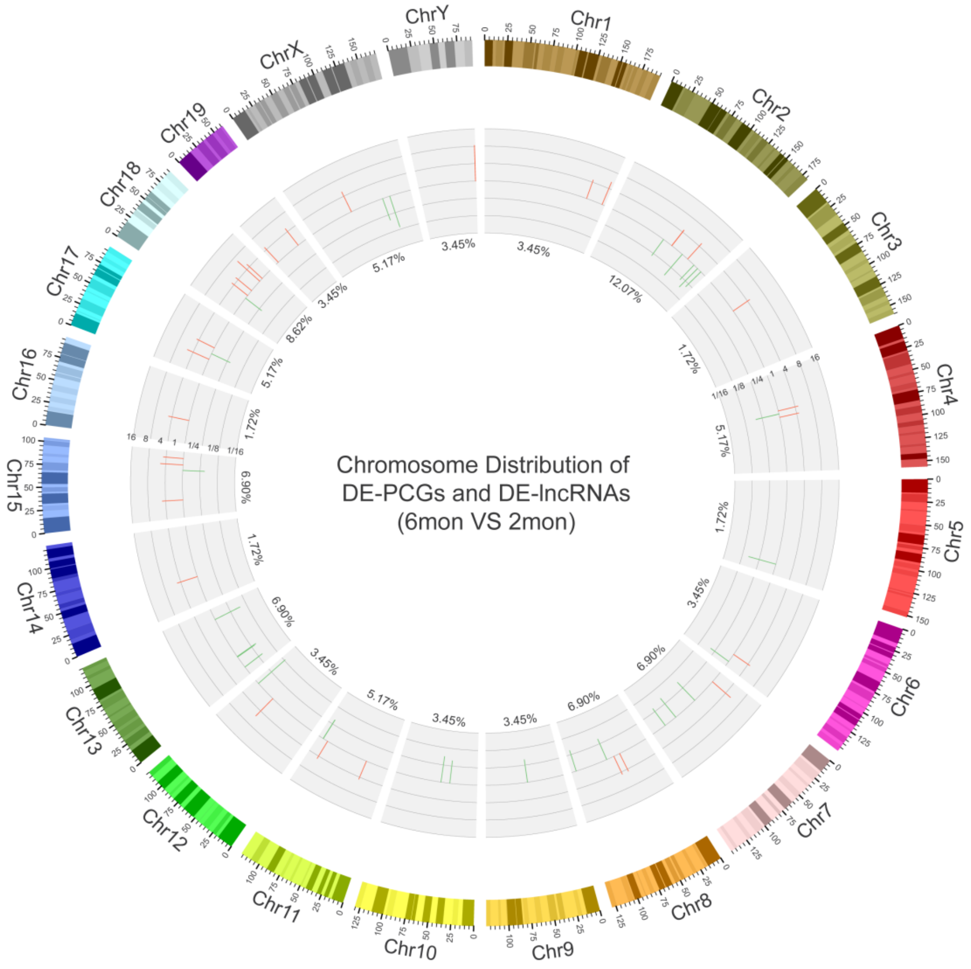


**Supplementary Figure 1. Chromosomal distribution of DE-PCGs and DE-lncRNAs of mature versus young adult mice.** The chromosomal distribution of 26 DE-PCGs and 32 DE-lncRNAs was identified. The proportion of DE-PCGs and DE-lncRNAs distributed on a given chromosome is marked in the corresponding areas. The outer circle depicts the ideograms of mouse chromosome. The internal circle shows the fold change of DE-PCGs or DE-lncRNAs with red and green bars representing up-regulation and down-regulation, respectively.

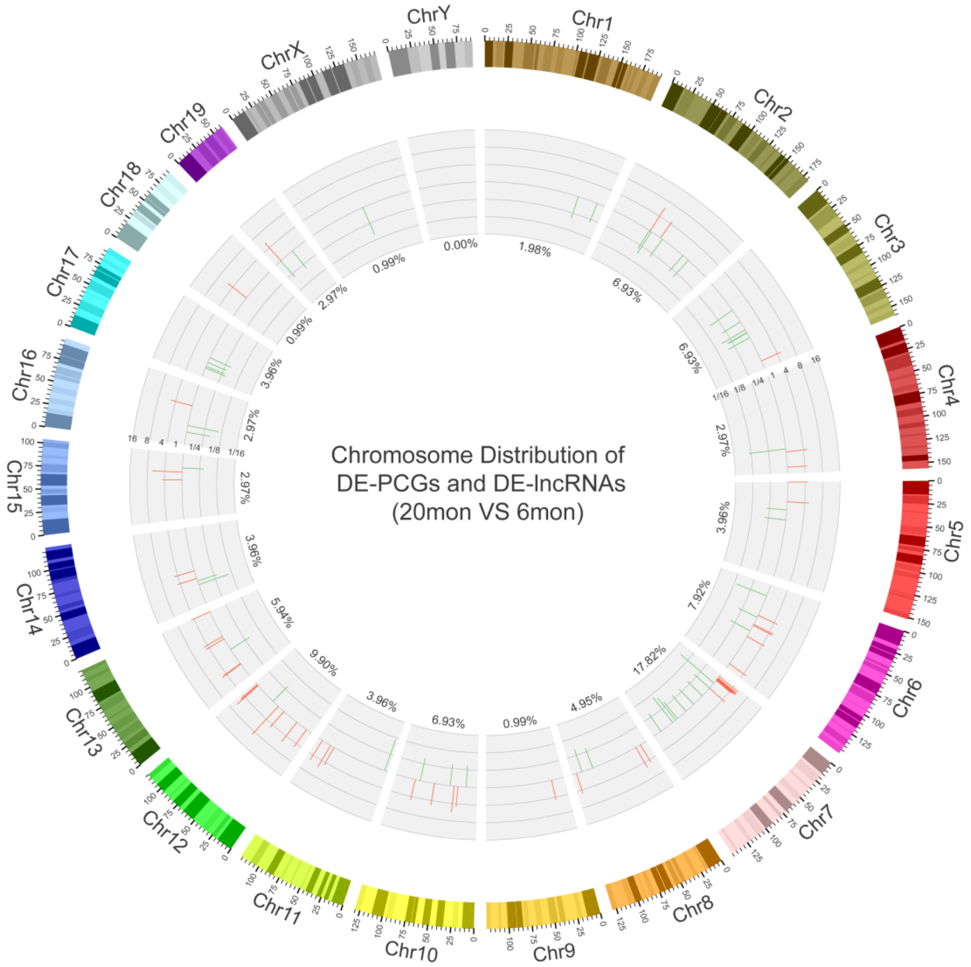


**Supplementary Figure 2. Chromosomal distribution of DE-PCGs and DE-lncRNAs of aged versus mature mice.** The chromosomal distribution of 39 DE-PCGs and 62 DE-lncRNAs was identified. The proportion of DE-PCGs and DE-lncRNAs distributed on a given chromosome is marked in the corresponding areas. The outer circle depicts the ideograms of mouse chromosome. The internal circle shows the fold change of DE-PCGs or DE-lncRNAs with red and green bars representing up-regulation and down-regulation, respectively.

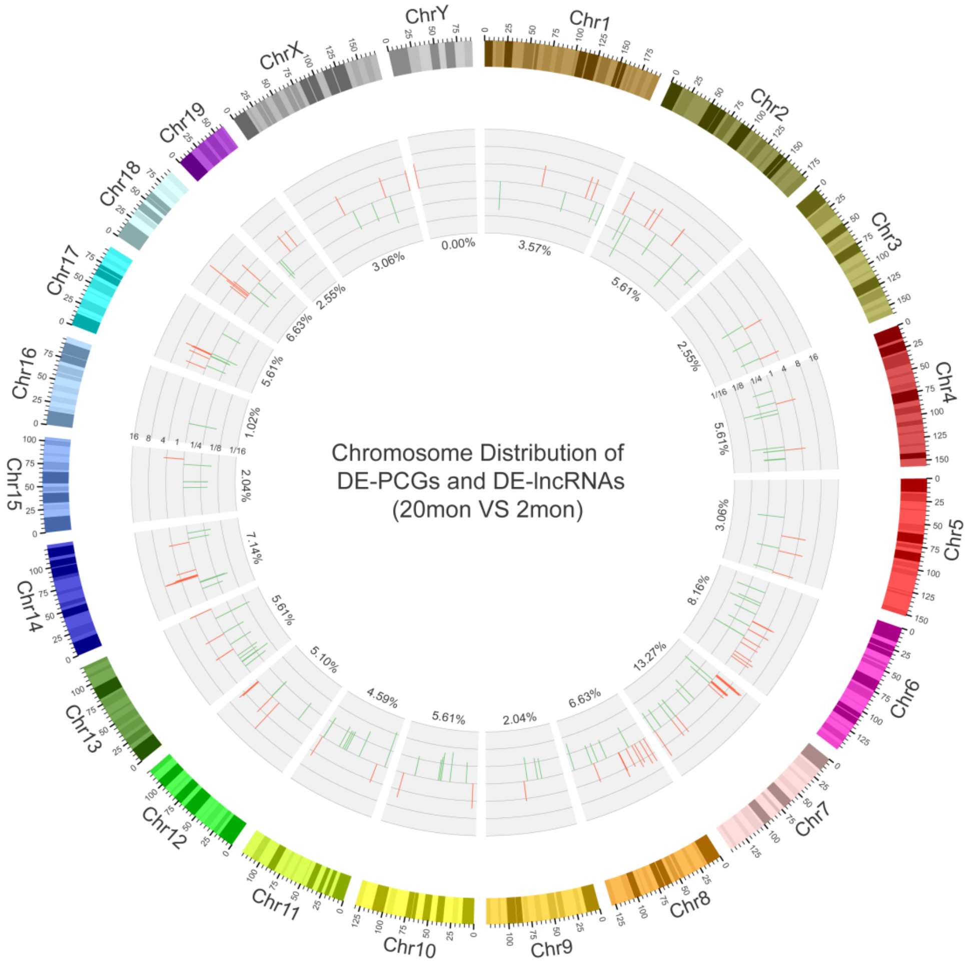


**Supplementary Figure 3. Chromosomal distribution of DE-PCGs and DE-lncRNAs of aged versus young adult mice.** The chromosomal distribution of 112 DE-PCGs and 84 DE-lncRNAs was identified. The proportion of DE-PCGs and DE-lncRNAs distributed on a given chromosome is marked in the corresponding areas. The outer circle depicts the ideograms of mouse chromosome. The internal circle shows the fold change of DE-PCGs or DE-lncRNAs with red and green bars representing up-regulation and down-regulation, respectively.

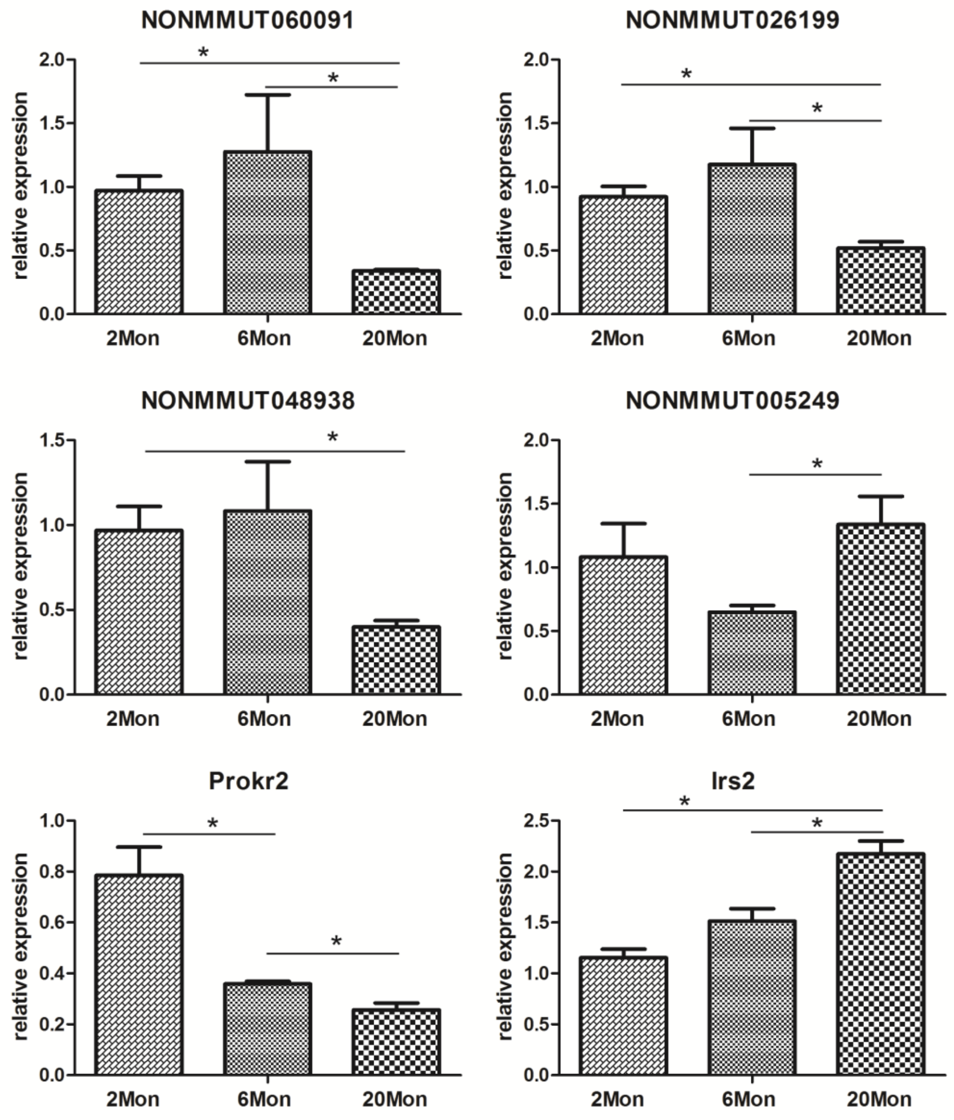


**Supplementary Figure 4. DE-mRNAs/lncRNAs' expression based on the microarray data.** The relative expression level of each mRNAs/lncRNAs was normalized. Bars represent S.E.M. ∗ $p$-value < 0.05.
